# Inhibition of the PLK1‐Coupled Cell Cycle Machinery Overcomes Resistance to Oxaliplatin in Colorectal Cancer

**DOI:** 10.1002/advs.202100759

**Published:** 2021-10-28

**Authors:** Zhaoliang Yu, Peng Deng, Yufeng Chen, Shini Liu, Jinghong Chen, Zihuan Yang, Jianfeng Chen, Xinjuan Fan, Peili Wang, Zerong Cai, Yali Wang, Peishan Hu, Dezheng Lin, Rong Xiao, Yifeng Zou, Yan Huang, Qiang Yu, Ping Lan, Jing Tan, Xiaojian Wu

**Affiliations:** ^1^ Department of Colorectal Surgery The Sixth Affiliated Hospital Sun Yat‐sen University Guangzhou Guangdong 510655 P. R. China; ^2^ Sun Yat‐sen University Cancer Center State Key Laboratory of Oncology in South China Collaborative Innovation Center of Cancer Medicine Guangzhou Guangdong 510060 P. R. China; ^3^ Guangdong Provincial Key Laboratory of Colorectal and Pelvic Floor Diseases Guangdong Institute of Gastroenterology The Sixth Affiliated Hospital Sun Yat‐sen University Guangzhou Guangdong 510655 P. R. China; ^4^ Department of Pathology The Sixth Affiliated Hospital Sun Yat‐sen University Guangzhou Guangdong 510060 P. R. China; ^5^ Department of Endoscopic Surgery The Sixth Affiliated Hospital Sun Yat‐sen University Guangzhou Guangdong 510060 P. R. China; ^6^ Department of Biomedical Sciences City University of Hong Kong Hong Kong SAR 999077 China; ^7^ Cancer and Stem Cell Biology Program Duke‐NUS Medical School Singapore 169857 Singapore; ^8^ Genome Institute of Singapore A*STAR Singapore 138672 Singapore; ^9^ Affiliated Cancer Hospital and Institute of Guangzhou Medical University Guangzhou Guangdong 510095 P. R. China

**Keywords:** cell division cycle 7, colorectal cancer, MYC, oxaliplatin, polo‐like kinase 1

## Abstract

Dysregulation of the cell cycle machinery leads to genomic instability and is a hallmark of cancer associated with chemoresistance and poor prognosis in colorectal cancer (CRC). Identifying and targeting aberrant cell cycle machinery is expected to improve current therapies for CRC patients. Here,upregulated polo‐like kinase 1 (PLK1) signaling, accompanied by deregulation of cell cycle‐related pathways in CRC is identified. It is shown that aberrant PLK1 signaling correlates with recurrence and poor prognosis in CRC patients. Genetic and pharmacological blockade of PLK1 significantly increases the sensitivity to oxaliplatin in vitro and in vivo. Mechanistically, transcriptomic profiling analysis reveals that cell cycle‐related pathways are activated by oxaliplatin treatment but suppressed by a PLK1 inhibitor. Cell division cycle 7 (CDC7) is further identified as a critical downstream effector of PLK1 signaling, which is transactivated via the PLK1‐MYC axis. Increased CDC7 expression is also found to be positively correlated with aberrant PLK1 signaling in CRC and is associated with poor prognosis. Moreover, a CDC7 inhibitor synergistically enhances the anti‐tumor effect of oxaliplatin in CRC models, demonstrating the potential utility of targeting the PLK1‐MYC‐CDC7 axis in the treatment of oxaliplatin‐based chemotherapy.

## Graphical abstract

Hyperactivity of PLK1‐MYC‐CDC7 axis correlates with recurrence and poor prognosis in colorectal cancer. Genetic and pharmacological blockade of this axis significantly increases the sensitivity to oxaliplatin in vitro and in vivo, providing potential clinical advantage in using PLK1 or CDC7 inhibitor in combination with chemotherapy regimens in treatment of CRC patients.

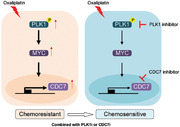

## 1 Introduction

CRC is one of the most common cancers worldwide and has a high recurrence rate.^[^ ^1^ ^]^ Tumor recurrence along with chemoresistance remains the main reason for CRC‐associated mortality. Therefore, there is an unmet clinical demand to understand the molecular oncogenic events leading to chemoresistance and explore new therapeutic strategies to improve patient survival. Disruption of the cell cycle machinery by genetic alterations has emerged as a characteristic feature of CRC, leading to genomic instability and chemoresistance,^[^ ^2^, ^3^ ^]^ highlighting that targeting aberrant cell cycle‐related pathways may improve the outcome of chemotherapy and overcome chemoresistance in CRC. To date, several small molecule inhibitors targeting cell cycle checkpoints have been developed for clinical applications, including PLK1 inhibitors, Aurora kinase inhibitors, and CDK inhibitors.^[^ ^4^, ^5^, ^6^ ^]^ Although an increasing number of clinical trials have shown that cell cycle checkpoint inhibitors could be an effective treatment option for relapsed or refractory cancer patients,^[^ ^7^, ^8^ ^]^ the clinical utilization of these inhibitors has been hindered due to limited successes in CRC.

PLK1 is a serine/threonine‐protein kinase, that is involved in multiple stages of cell cycle progression in mammals.^[^ ^9^ ^]^ Dysregulation of PLK1 is highly frequent in various malignancies and is correlated with poor prognosis in many cancers.^[^ ^10^ ^]^ Previous studies have shown that PLK1 is overexpressed in CRC tumors compared with normal tissue and which is correlated with disease progression, including adverse invasion, metastasis, and prognosis.^[^ ^11^, ^12^, ^13^ ^]^ Moreover, our previous study showed that PLK1 can stabilize MYC activation to confer cellular oncogenic transformation and tumor stem cell activation, indicating a potential functional link of PLK1 with MYC in oncogenesis.^[^ ^14^ ^]^ However, two other studies showed that PLK1 functions as a tumor suppressor in tumorigenesis by inducing chromosomal instability.^[^ ^15^, ^16^ ^]^ Thus, the role of PLK1 in CRC remains controversial, hindering the clinical implementation of PLK1 inhibitors in CRC.^[^ ^15^ ^]^ Furthermore, the anti‐tumor effect of PLK1 inhibitors in CRC remains a clinical challenge due to the diverse clinical outcomes and lack of biomarkers, suggesting that there is a clinical demand to further investigate the potential mechanisms of PLK1 inhibitors in CRC.^[^ ^17^, ^18^ ^]^

Several PLK1 inhibitors have been developed based on the biological function and structure of PLK1 for clinical trials as cancer therapies.^[^ ^4^ ^]^ One of these inhibitors, volasertib was designated “breakthrough therapy” for acute myelogenous leukaemia by the Food and Drug Administration (FDA) in 2013.^[^ ^19^, ^20^ ^]^ Oxaliplatin‐based chemotherapy is one of the standard treatment strategies for late‐stage colon cancer patients.^[^ ^21^, ^22^ ^]^ However, resistance to chemotherapy is a crucial factor contributing to poor patient survival.^[^ ^23^, ^24^ ^]^ A recent phase II clinical trial showed that 11% of patients with platinum‐resistant or platinum‐refractory ovarian cancer treated with volasertib had a prolonged progression‐free survival of more than 1 year.^[^ ^8^ ^]^ Additionally, another phase I clinical trial indicated that two platinum‐refractory head and neck squamous cell carcinoma patients treated with volasertib achieved a complete response or partial response, respectively.^[^ ^18^ ^]^ These studies suggest that the combination of a PLK1 inhibitor with platinum‐containing chemotherapy could be an attractive therapeutic strategy to overcome chemoresistance in CRC.

This study showed that aberrant activation of PLK1 signaling, which causes a dysfunctional cell cycle, was correlated with poor prognosis and tumor recurrence in CRC. Genetic and pharmacological inhibition of PLK1 overcame resistance to oxaliplatin in vitro and in vivo. Importantly, we identified that CDC7 is a key downstream effector of PLK1 signaling through MYC transactivation, which confers resistance to oxaliplatin, as evidenced in both in vitro and in vivo models. Our findings collectively provide a potential therapeutic strategy for relapsed/refractory CRC with immediate clinical implications.

## 2 Results

### 2.1 Dysfunction of the PLK1 Signaling Pathway Promotes Tumorigenesis of CRC

**Figure 1:**
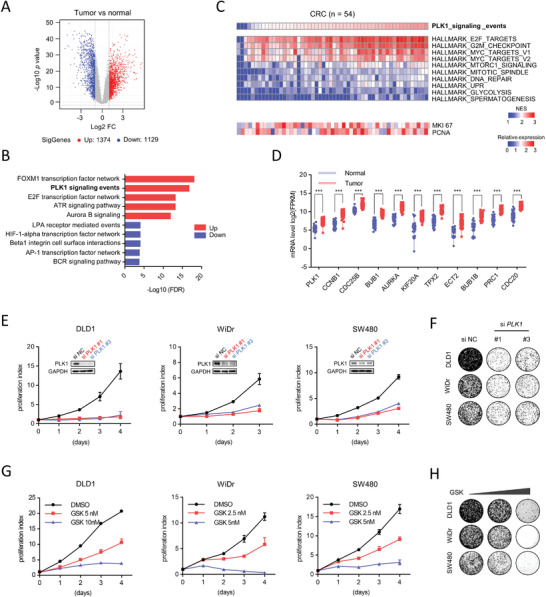
Dysfunction of PLK1 signaling pathway promotes tumorigenesis of CRC. A) The differentially expressed genes in 54 pairs of human colorectal tumor versus matched normal mucosa are shown in the volcano plot. B) The top five up/down enrichment pathways in tumors versus matched normal mucosa. C) Heatmap of NES values in GSEA analysis of tumors compared with matched normal mucosa using hallmark gene sets. 54 patients were listed according to the NES values in GSEA analysis with PLK1 signaling gene set, top 10 hallmark gene sets are shown, and relative expression of MKI67 and PCNA is shown. D) Relative mRNA levels of PLK1 signaling genes in CRC samples and matched normal colon tissues. E) Cell viability of indicated cancer cell lines after silencing PLK1 or applying a control. F) Colony formation assay in cells treated with siPLK1 knockdown after 12 days culture. G) Cell viability of indicated cancer cell lines after treatment with different doses of PLK1 inhibitor (GSK461364), with applying DMSO as a control. H) Colony formation assay in cells treated with indicated concentration of GSK461364 in DLD1 (5, 10 nm) and in WiDr and SW480 (2.5, 5 nm). Cells were stained with crystal violet after 12 days. Error bars represent ± SD (E,G). Data are representative of three independent experiments (E–H).

Deregulation of the cell cycle is a hallmark of cancer and is associated with loss of cell cycle control.^[^ ^2^ ^]^ To characterize dysfunctional genes related to the cell cycle in CRC, we interrogated genome‐wide profiling analysis of differentially expressed genes in 54 pairs of human colorectal tumor with matched normal mucosa from our previously published dataset (GSE10972 and GSE74602) (**Figure** **1**A). Gene set enrichment analysis (GSEA) indicated that several cell cycle related pathways were highly enriched in tumor compared to matched normal tissues, including PLK1 signaling, E2F transcription factor network, and Aurora A/B signaling (Figure 1B). Notably, a cluster of canonical cell cycle related pathways was significantly activated in most of the tumors, including MYC target genes, G2/M checkpoint genes and E2F target genes (Figure 1C). Intriguingly, tumors with enriched PLK1 signaling were positively correlated with deregulated cell cycle pathways (*p* < 0.001; *R* ^2^ > 0.5; Figure S1A, Supporting Information) but weakly correlated with MKI67 or PCNA (MKI67, *p* = 0.040, *R* ^2^ = 0.153; PCNA, *p* = 0.327, *R* ^2^ = 0.019), and there was no correlation between MKI67 or PCNA and cell cycle pathways (*p* > 0.05, *R* ^2^ < 0.2; Figure S1B, Supporting Information), suggesting that increased PLK1‐coupled cell cycle pathways are not due to an increase in cell proliferation. Moreover, all the genes involved in PLK1 signaling were highly expressed in tumors compared to matched normal mucosa (Figure 1D). Highly enriched PLK1 signaling was further confirmed in pairs of colorectal tumors with matched normal mucosa from the CIT cohort (GSE39582) and TCGA cohort (COAD & READ) (Figure S1C–F, Supporting Information). These results suggested that the PLK1‐coupled cell cycle machinery may function as a key event to promote tumorigenesis in CRC. A functional study showed that depletion of PLK1 by two individual small interfering RNAs (siRNAs) significantly inhibited the proliferation and colony formation of CRC cells (Figure 1E,F), which was further confirmed by using two PLK1 inhibitors (GSK461364 and volasertib) (Figure 1G,H; Figure S1G,H, Supporting Information). Thus, these findings support that PLK1 promotes tumorigenesis in CRC, and targeting PLK1 signaling could be a potential therapeutic strategy against CRC.

### 2.2 Hyperactivity of PLK1 is Associated with Recurrence and Poor Prognosis in CRC

**Figure 2:**
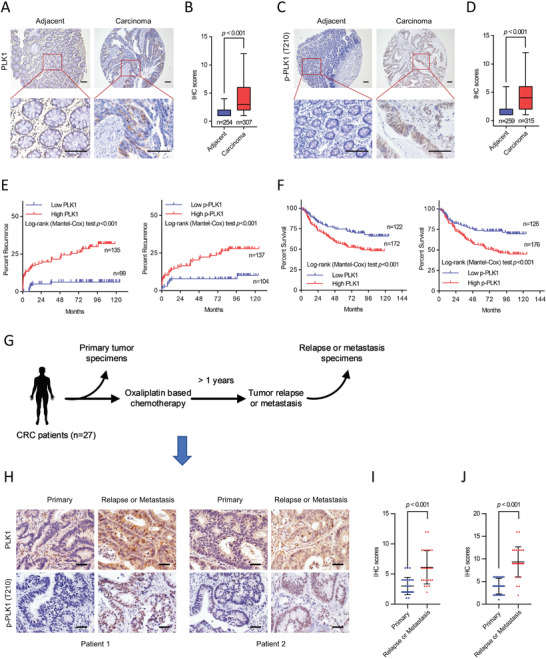
Hyperactivity of PLK1 is associated with CRC recurrence and poor prognosis. A) Representative IHC staining of PLK1 in CRC and normal tissues, and high‐ magnification images of representative IHC staining. B) IHC scores of PLK1 expression in tumor tissue versus adjacent tissue. C) Representative IHC staining of p‐PLK1 (T210) in CRC and normal tissues, and high‐ magnification images of representative IHC staining. Scale bar = 100 µm (A,C). D) IHC scores of p‐PLK1 expression in tumor tissue versus adjacent tissue. *p* values were determined by two‐tailed Student's *t‐*test (B,D). E) Kaplan–Meier curves of recurrence time in CRC patients according to high and low PLK1/p‐PLK1 expression. F) Kaplan–Meier curves of overall survival rates in CRC patients according to high and low PLK1/p‐PLK1 expression. G) Schematic diagram of collecting primary tumor specimens and relapse or metastasis specimens from the same patient underwent oxaliplatin based chemotherapy. H) Representative IHC images for PLK1 and p‐PLK1 (T210) in primary and relapse or metastasis CRC tissues. Scale bar = 50 µm. I) IHC scores of PLK1 expression in primary tumor tissue versus relapse or metastasis tumor tissue. J) IHC scores of p‐PLK1 expression in primary tumor tissue versus relapse or metastasis tumor tissue. IHC scores were determined by the intensity score and the proportion of area positively stained tumor cells. *p* values were determined by two‐tailed paired Student's *t‐*test (I,J).

Although overexpression of PLK1 is frequently found in various human cancers, the evidence for the clinical and prognostic relevance of PLK1 is controversial in different cancer types.^[^ ^10^, ^15^ ^]^ To investigate the clinical significance of PLK1 in CRC, we examined the protein level of PLK1 in CRC patient samples by using a tissue microarray (TMA) on colon or rectal tumor tissues obtained from 343 CRC patients (Table S1, Supporting Information). Consistent with previous studies,^[^ ^12^, ^13^ ^]^ PLK1 was significantly upregulated in CRC tumors compared to matched normal counterpart colon tissues (*p* < 0.001; **Figure** **2**A,B), which was further confirmed using databases from four published studies (Figure S2, Supporting Information). In addition, the level of p‐PLK1 (T210) was positively correlated with PLK1 expression (*p* < 0.001; Figure 2C,D). Additionally, a high level of PLK1/p‐PLK1 was correlated with a higher tumor recurrence rate (*p* < 0.001; Figure 2E) and a shorter overall survival (OS) time (*p* < 0.001; Figure 2F). Moreover, PLK1 could be used as an independent prognostic factor for tumor recurrence in CRC according to univariate and multivariate analyses (Table S2, Supporting Information). A previous study showed that the PLK1 inhibitor volasertib could improve clinical outcomes in platinum‐resistant cancers.^[^ ^8^ ^]^ To determine whether PLK1 activation is correlated with CRC progression after oxaliplatin based chemotherapy, we examined levels of PLK1 and p‐PLK1 in a set of CRC tissues from primary and relapse/metastasis samples (Figure 2G). Higher levels of PLK1/p‐PLK1 were detected in relapsed/metastatic CRC tissues than in matched primary CRC tissues (Figure 2H–J). These data indicated that hyperactivity of PLK1 is not only associated with poor prognosis, but also with tumor relapse/metastasis, supporting PLK1 may confer resistance to oxaliplatin‐based chemotherapy in CRC.

### 2.3 . The Efficacy of Oxaliplatin is Enhanced by PLK1 Inhibition In Vitro and In Vivo

**Figure 3:**
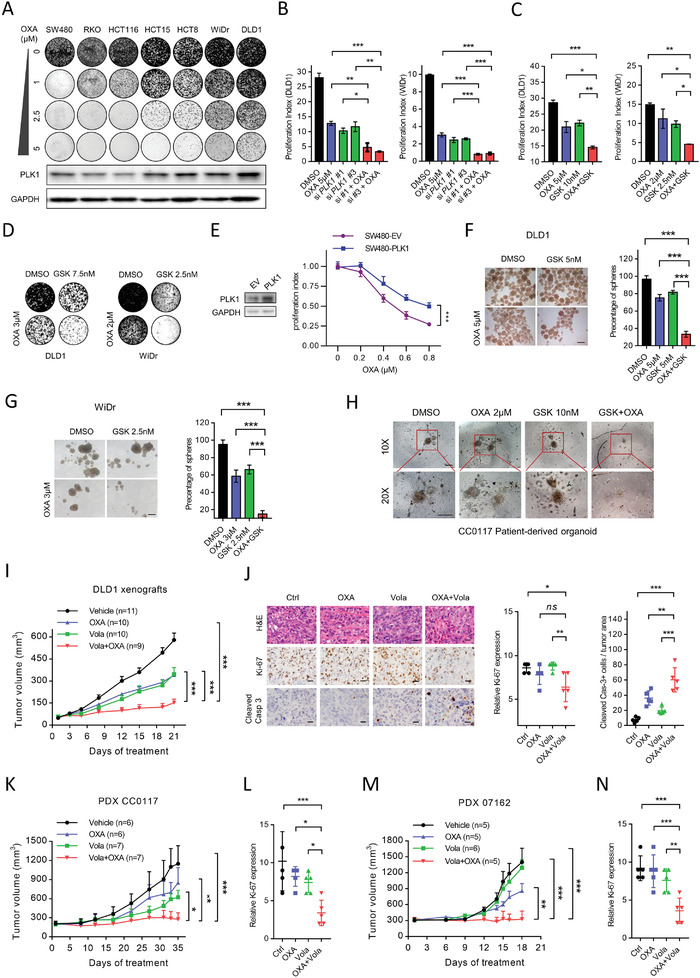
PLK1 inhibition overcomes the resistance to oxaliplatin in vitro and in vivo. A) Colony formation assay of indicated cancer cell lines treated with different dosages of oxaliplatin. Cells were stained with crystal violet after 12 days of cell culture. B) Cell viability of indicated cancer cell lines treated with 2 siRNAs targeting PLK1, oxaliplatin, or both. Cell viability was determined on day 1 and day 5 after treatment and proliferation index was calculated as fold change of cell viability. Error bars represent ± SD. C) Cell viability of indicated cancer cell lines treated with PLK1 inhibitor (GSK461364) at the presence or absence of oxaliplatin for 5 days. Error bars represent ± SD. D) Colony formation assay in cells treated PLK1 inhibitor (GSK461364), oxaliplatin, or combination at 12 days. E) Effects of PLK1 overexpression in SW480 cells on oxaliplatin sensitivity, assessed by growth curves. F) Representative image of tumor sphere formation assay in DLD1 cells treated with GSK461364, oxaliplatin, or combination (left) after 10 days of cell culture. Scale bar = 500 µm. Relative tumor spheres in cells treated with GSK461364, oxaliplatin, or combination (right). G) Representative image of tumor sphere formation assay in WiDr cells treated with GSK461364, oxaliplatin, or combination (left) after 10 days of cell culture. Scale bar = 500 µm. Relative tumor spheres in cells treated with GSK461364, oxaliplatin, or combination (right). H) Representative image of PDO CC0117 treated with GSK461364, oxaliplatin, or combination after 16 days culture. Scale bar = 200 µm. I) The growth curve of tumor volume in vivo efficacy of oxaliplatin and PLK1 inhibitor (volavertib) in DLD xenografts. Error bars represent ± SEM. J) Representative image of hematoxylin‐eosin staining (HE) and IHC of Ki‐67 and cleaved‐caspase 3 in DLD1 xenograft tumors (left). Scale bar = 20 µm. Relative Ki‐67 and relative cleaved‐caspase 3 positive cells expression in four groups of DLD1 xenograft tumors (right). K) The growth curve of tumor volume in vivo efficacy of oxaliplatin and volasertib in PDX CC0117 model. Error bars represent ± SEM. L) Relative Ki‐67 expression in four groups of PDX CC0117 model. M) The growth curve of tumor volume in vivo efficacy of oxaliplatin and volasertib in PDX 07162 model. N) Relative Ki‐67 expression in four groups of PDX 07162 model. Error bars represent ± SEM. Data are representative of three independent experiments (A–G). *p< 0.05, **p< 0.01, ***p< 0.001, one‐way ANOVA with Dunnett's multiple comparisons test (B–D,F,G,J,L,N), and two‐way ANOVA with Dunnett's multiple comparisons test (I,K,M).

Resistance to oxaliplatin‐based chemotherapy represents a significant clinical problem in CRC, resulting in tumor recurrence. Elevated PLK1 expression is correlated with the recurrence of CRC, suggesting that PLK1 may confer resistance to chemotherapy in CRC. To determine whether PLK1 overexpression is associated with chemoresistance in CRC, we examined the correlation between the PLK1 expression level and the cytotoxic effect of oxaliplatin in a panel of colon cancer cell lines. The data showed that oxaliplatin‐induced growth inhibition was much more effective in CRC cells with low PLK1 expression than in those with high PLK1 expression, as measured by colony formation assay (**Figure** **3**A). In addition, knockdown of PLK1 with siRNA significantly enhanced the effect of oxaliplatin on proliferation and colony formation (Figure 3B; Figure S3A, Supporting Information), which was further confirmed with PLK1 inhibitors (Figure 3C,D; Figure S3B, Supporting Information). Moreover, ectopically expressing PLK1 confers resistance to oxaliplatin in SW480 cells, supporting the important role of PLK1 in oxaliplatin resistance in CRC (Figure 3E). Cancer stem cells (CSCs) have been shown to display enhanced resistance to chemotherapy, contributing to tumor recurrence.^[^ ^25^, ^26^ ^]^ We next investigated the role of PLK1 on CSCs in CRC. Genetic and pharmacological inhibition of PLK1 showed a strong combinatorial effect with oxaliplatin to block tumorsphere formation, indicating the critical role of PLK1 in tumor‐initiation capability (Figure 3F,G; Figure S3C, Supporting Information). Similar results were found with the other two PLK1 inhibitors (volasertib and BI2536), which significantly decreased tumorsphere formation (Figure S3D, Supporting Information). More importantly, the combination of the PLK1 inhibitor GSK and oxaliplatin effectively inhibited the growth of patient‐derived organoids from CRC patient CC0117 (Figure 3H), suggesting that PLK1 inhibition may enhance the sensitivity of CRC patients to oxaliplatin.

To further confirm this combination effect in vivo, we next examined the anti‐tumor efficacy of combination treatment with a PLK1 inhibitor and oxaliplatin in a DLD1‐derived xenograft mouse model. The combination of oxaliplatin and volasertib led to a significantly greater anti‐tumor effect (Figure 3I), which showed remarkably decreased Ki‐67 and increased cleaved‐caspase 3 by immunohistochemistry (IHC) analysis (Figure 3J). Furthermore, in two CRC patient‐derived xenograft (PDX) models in which high levels of PLK1 and p‐PLK1 were detected by IHC analysis (Figure S3E, Supporting Information), drug combination treatment resulted in complete suppression of tumor growth compared to single treatment (Figure 3K,M) and the proliferation marker Ki‐67 was significantly decreased (Figure 3L,N). These findings collectively demonstrate the potential clinical implications of PLK1 inhibitors in combination with oxaliplatin‐based chemotherapy regimens in the treatment of CRC patients.

### 2.4 CDC7 is a Critical Downstream Effector of PLK1 through the PLK1‐MYC Axis

**Figure 4:**
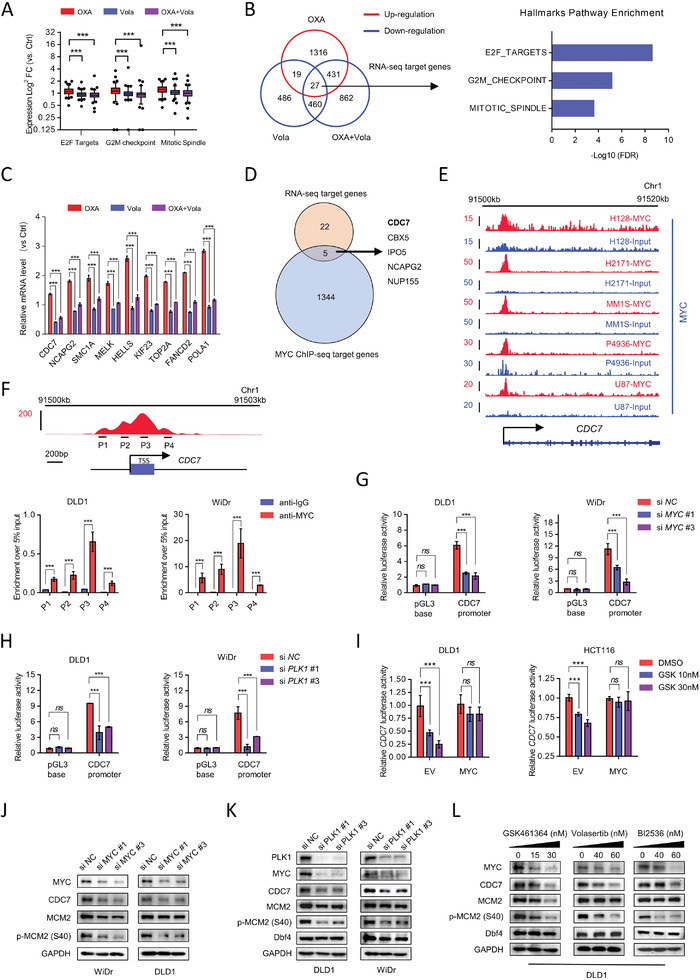
PLK1 inhibition blocks CDC7 transactivation via MYC signaling. A) Box plots showing expression changes of cell cycle pathway genes in DLD1 cells treated with 5 µm oxaliplatin, 40 nm volasertib, or combination for 48 h. The vertical axis represents the log_2_ (TPM) in the indicated genotype group. B) Venn diagram showing up‐ and down‐regulated genes (adjust *p* < 0.05) by oxaplatin, volasertib, or combination compared with control (left) and the hallmark pathway analysis of 27 target genes (right). C) RT‐qPCR analysis of candidated genes in DLD1 treated with 5 µm oxaplatin, 40 nm volasertib, or combination. D) Venn diagram showing the RNA‐seq target genes and the MYC ChIP‐seq target genes. E) Track view of MYC CHIP‐seq density profile on CDC7 genomic region from published datasets. The *x* axis shows genomic position. The *y* axis shows signal strength of MYC binding. F) Schematic diagram of the ChIP primer (P1–P4) locations across the CDC7 promoter region (up). Chromatin extracts from DLD1 and WiDr cells were subjected to ChIP using anti‐MYC antibody or normal IgG, genomic regions of the CDC7 promoter were tested for enrichment of MYC binding (down). Data are presented relative to input and shown as mean ± SD of technical triplicates. G) CDC7 promoter luciferase reporter was transfected into DLD1 and WiDr cells infected with control siRNA or MYC siRNA, and luciferase activity was measured 48 h after transfection. pGL3‐base is the empty vector control for CDC7 promoter reporter. Data are presented relative to Renilla readings and shown as mean ± SD of biological triplicates. H) CDC7 promoter luciferase reporter was transfected into DLD1 and WiDr cells infected with control siRNA or siPLK1, and luciferase activity was measured 48 h after transfection. Data are presented relative to Renilla readings and shown as mean ± SD of biological triplicate. I) DLD1 and HCT116 cells were infected with retroviral constructs expressing empty vector and MYC. CDC7 promoter luciferase reporter was transfected into the cells and the cells were treated with DMSO or PLK1 inhibitor (GSK461364) for 24 h. Data are presented relative to Renilla readings and shown as mean ± SD of biological triplicate. J) Immunoblot analysis of DLD1 and WiDr cells infected with two siRNAs targeting MYC. Samples were collected at 72 h after siRNA transfection. K) Immunoblot analysis of DLD1 and WiDr cells infected with two siRNAs targeting PLK1. Samples were collected at 72 h after siRNA transfection. L) Immunoblot analysis of DLD1 cells treated with PLK1 inhibitors for 48 h. *p < 0.05, **p < 0.01, ***p < 0.001, and two‐tailed Student's *t‐*test (C,F–I).

To investigate the underlying mechanism of the synergistic effect of oxaliplatin and PLK1 inhibitors, we first performed RNA‐sequencing (RNA‐seq) in DLD1 cells treated with oxaliplatin in the presence or absence of volasertib. GSEA showed that cell cycle‐related pathways, including mitotic spindle, E2F targets, and G2M checkpoint, were activated in oxaliplatin treatment but inhibited in volasertib and combination treatment (**Figure** **4**A; Figure S4A, Supporting Information), suggesting that PLK1 is a key mediator of cell‐cycle deregulation in CRC. Recent studies demonstrated that the upregulation of drug tolerance‐related genes caused by chemo‐drug contributed to chemoresistance,^[^ ^27^ ^]^ prompting us to hypothesize that PLK1 inhibition may suppress a cluster of oxaliplatin‐induced genes. Transcriptomic profiling analysis showed that a total of 27 oxaliplatin‐activated genes were downregulated in the volasertib and combination groups (Figure 4B). Hallmarks pathway enrichment showed that most of these genes were related to cell cycle regulation, including CDC7, MELK, TOP2A, and SMC1A (Figure 4B; Figure S4B, Supporting Information), which was further confirmed by real‐time quantitative PCR (RT‐qPCR) analysis (Figure 4C). In addition, GSEA showed that MYC target genes were remarkably suppressed by PLK1 inhibition (Figure S4A,C, Supporting Information), which is consistent with previous studies that showed that PLK1 confers oncogenic transformation by stabilizing MYC activation.^[^ ^14^, ^28^, ^29^ ^]^ To determine whether these downstream targets were directly regulated by MYC transactivation, we examined the MYC ChIP‐seq database in various cancer cells. We identified MYC targets by profiling the MYC ChIP‐seq dataset, showing that a total of five genes overlapped in both RNA‐seq targets and MYC targets (Figure 4D). Among these genes, CDC7, which encodes CDC7, is involved in the maintenance of DNA replication forks and the DNA damage response,^[^ ^30^ ^]^ and is considered a key downstream effector of PLK1 signaling. CDC7 is a serine/threonine kinase that plays a critical role in the initiation of DNA replication by phosphorylating the MCM 2–7 complex, which is required for genome duplication.^[^ ^30^ ^]^ As expected, both PLK1 inhibitor and CDC7 inhibitor combined with oxaliplatin arrested tumor cells at G2/M phase (Figure S4D, Supporting Information). Moreover, phosphor‐histone H3, a mitotic phase marker, was dramatically increased in combination treatment, which was further confirmed by a synchronization assay (Figure S4E–G, Supporting Information), suggesting that decreased G2/M phase genes by combination treatment trigger mitotic entry. CDC7 is frequently overexpressed in various human cancers,^[^ ^31^, ^32^, ^33^ ^]^ making it an attractive therapeutic target for cancer treatment. However, the role of CDC7 in CRC patients and the molecular mechanism underlying tumorigenesis remain unclear. The MYC‐binding region is on the proximal promoter region of these five genes, including CDC7, suggesting that MYC may directly bind to the CDC7 promoter region to activate its transcription (Figure 4E; Figure S5A, Supporting Information). This was confirmed by ChIP‐quantitative PCR (ChIP‐qPCR), indicating that MYC directly binds to the promoter of CDC7 in CRC cells (Figure 4F). In addition, the luciferase reporter assay demonstrated that the luciferase intensity of the CDC7 gene promoter was significantly increased in control cells but significantly repressed by siRNA‐mediated MYC depletion (Figure 4G). Consistently, genetic and pharmacological inhibition of PLK1 repressed the transcription intensity of CDC7 (Figure 4H; Figure S5B, Supporting Information). Moreover, ectopically expressed MYC rescued the decreased CDC7 transcription level induced by the PLK1 inhibitor (Figure 4I), suggesting that PLK1 regulates CDC7 activity in an MYC‐dependent manner. MYC depletion dramatically suppressed CDC7 and its downstream target p‐MCM2 (S40), indicating the regulation of CDC7 by MYC (Figure 4J). Genetic and pharmacological inhibition of PLK1 was able to suppress MYC, CDC7, and p‐MCM2 expression (Figure 4K,L), further indicating that CDC7 is a key effector of PLK1‐MYC signaling.

### 2.5 CDC7 Overexpression is Associated with Poor Prognosis and Positively Correlated with PLK1‐MYC Signaling in CRC

**Figure 5:**
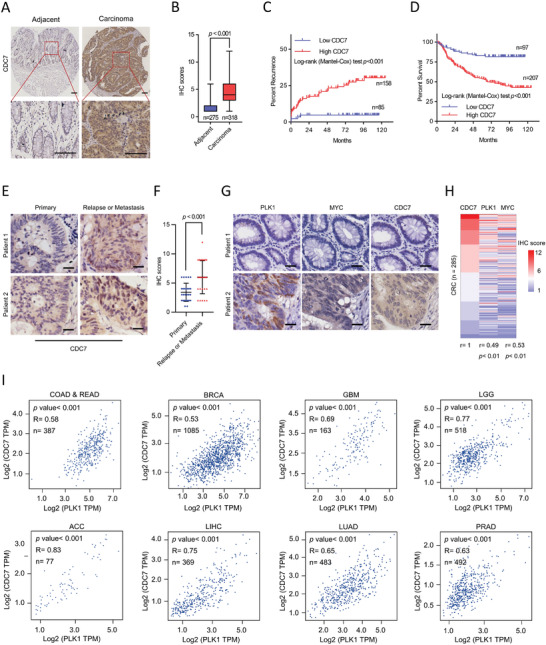
Aberrant PLK1‐MYC‐CDC7 signaling is associated with poor prognosis and recurrence in CRC. A) Representative IHC staining of CDC7 in CRC and normal tissues, and high‐ magnification images of representative IHC staining. Scale bar = 100 µm. B) IHC scores of CDC7 expression in tumor tissue versus adjacent tissue. *p* value was determined by two‐tailed Student's *t‐*test. C) Kaplan–Meier curves of recurrence time in CRC patients according to high and low CDC7 expression. D) Kaplan–Meier curves of overall survival rates in CRC patients according to high and low CDC7 expression. E) Representative IHC images for CDC7 in primary and relapse or metastasis CRC tissues. Scale bar = 100 µm. F) IHC scores of CDC7 expression in primary tumor tissue versus relapse or metastasis tumor tissue (*n* = 27). IHC scores were determined by the intensity score and the proportion of area positively stained tumor cells. *p* values were determined by two‐tailed paired Student's *t‐*test. G) Representative IHC staining of PLK1, MYC, and CDC7 in the same patients. Scale bar = 200 µm. H) Heatmap of IHC score (by Spearman relevance) between PLK1, MYC, and CDC7 in CRC specimens. I) Positive correlation (by Pearson's) between CDC7 and PLK1 in eight types of cancer. Data were derived from the TCGA dataset. COAD: colon adenocarcinoma, READ: rectum adenocarcinoma, BRCA: breast invasive carcinoma, GBM: glioblastoma multiforme, LGG: brain lower grade glioma, ACC: adrenocortical carcinoma, LIHC: liver hepatocellular carcinoma, LUAD: lung adenocarcinoma, and PRAD: prostate adenocarcinoma.

To evaluate the potential signaling of PLK1‐MYC‐CDC7 in CRC patients, we first investigated the clinical relevance of CDC7 in CRC. TMA analysis indicated that CDC7 was overexpressed in CRC tumors compared to matched normal counterpart colon tissues (*p* < 0.001; **Figure** **5**A,B). Overexpression of CDC7 was significantly correlated with a higher tumor recurrence rate (*p* < 0.001; Figure 5C), and shorter OS time (*p* < 0.001; Figure 5D). Moreover, higher levels of CDC7 were detected in relapsed/metastatic CRC tissues than in matched primary CRC tissues (Figure 5E,F). To determine the correlation between CDC7 and PLK1‐MYC signaling in CRC, we performed a correlation analysis between PLK1, MYC, and CDC7 expression in a cohort of CRC patients and found that PLK1 and MYC showed a strong positive correlation with the expression of CDC7 (Figure 5G,H). Furthermore, the pan‐cancer analysis also confirmed this positive correlation using TCGA dataset (Figure 5I). Together, these findings demonstrate that CDC7 is a key downstream effector of PLK1 via MYC transcriptional activity, and may be a potential therapeutic target for CRC with aberrant activation of PLK1‐MYC signaling.

### 2.6 CDC7 Inhibition Significantly Enhances the Anti‐Tumor Effect of Oxaliplatin In Vitro and In Vivo

**Figure 6:**
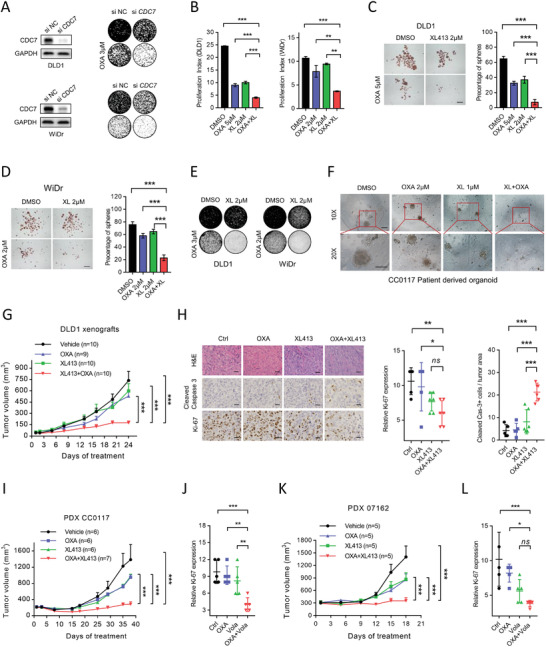
CDC7 inhibition overcomes the resistance to oxaliplatin in vitro and in vivo. A) Colony formation assay in cells treated with siRNA siCDC7, oxaliplatin, or both after 12 days of cell culture. B) Cell viability of indicated cancer cell lines treated with CDC7 inhibitor (XL413) in the presence or absence of oxaliplatin for 5 days. Error bars represent ± SD. C) Representative image of tumor sphere formation assay in DLD1 cells treated with XL413, oxaliplatin, or combination after 10 days of cell culture (left). Scale bar = 500 µm. Relative tumor spheres in cells treated with XL413, oxaliplatin, or combination (right). D) Representative image of tumor sphere formation assay in WiDr cells treated with XL413, oxaliplatin, or combination after 10 days of cell culture (left). Scale bar = 500 µm. Relative tumor spheres in cells treated with XL413, oxaliplatin, or combination (right). E) Colony formation assay in cells treated with CDC7 inhibitor (XL413), oxaliplatin, or combination. Cells were stained with crystal violet after 12 days. F) Representative image of PDO CC0117 treated with XL413, oxaliplatin, or combination after 16 days culture. Scale bar = 200 µm. G) The growth curve of tumor volume in vivo efficacy of oxaliplatin and CDC7 inhibitor (XL413) in DLD xenografts. Error bars represent ± SEM. H) Representative image of HE and IHC of Ki‐67 and cleaved‐caspase 3 in DLD1 xenograft tumors (left). Scale bar = 20 µm. Relative Ki‐67 expression and relative cleaved‐caspase 3 positive cells in four groups of DLD1 xenograft tumors (right). I) The growth curve of tumor volume in vivo efficacy of oxaliplatin and volasertib in PDX CC0117 model. J) Relative Ki‐67 expression in four groups of PDX CC0117 model. K) The growth curve of tumor volume in vivo efficacy of oxaliplatin and volasertib in PDX 07162 model. L) Relative Ki‐67 expression in four groups of PDX 07162 model. Data are representative of three independent experiments (A–E). *p < 0.05, **p < 0.01, ***p < 0.001, one‐way ANOVA with Dunnett's multiple comparisons test (B–D,H,J,L), and two‐way ANOVA with Dunnett's multiple comparisons test (G,I,K).

To investigate the crucial role of CDC7 as a downstream effector of PLK1, we depleted CDC7 with siRNA and found that CDC7 depletion dramatically inhibited colony formation in CRC cells treated with oxaliplatin (**Figure** **6**A). Consistently, pharmacological inhibition with CDC7 inhibitors (XL413 or PHA767491) overcomes resistance to oxaliplatin in cells, assayed using proliferation, colony formation, and tumorsphere formation CRC cells (Figure 6B–E; Figure S6A–C, Supporting Information), which recapitulated the phenotypes of PLK1 inhibitor. Similar results were shown in the CC0117 patient‐derived organoid model (Figure 6F), suggesting that the preclinical drug XL413 may enhance the anti‐tumor effect of oxaliplatin in the clinic. To further validate these findings in vivo, we examined the efficacy of a CDC7 inhibitor and oxaliplatin in a DLD1‐derived xenograft mouse model and two PDX models. The combination of oxaliplatin and XL413 significantly inhibited xenograft tumor growth (Figure 6G), which showed remarkably decreased Ki‐67 and increased cleaved‐caspase 3 in IHC analysis (Figure 6H). Furthermore, drug combination effects were observed to significantly inhibit tumor growth in two CRC PDX models (Figure 6I–L). These findings collectively confirm that CDC7 is a crucial downstream effector of PLK1 for cell survival upon oxaliplatin treatment and that targeting the PLK1‐MYC‐CDC7 axis can enhance oxaliplatin‐based chemotherapy regimens in CRC.

## 3 Discussion

Although oxaliplatin‐based chemotherapy is the standard treatment of CRC, numerous patients inevitably develop resistance and recurrence. Thus, identifying the key molecular driver events associated with chemoresistance will allow for new therapeutic strategies, which are essential for improving patient survival. In this study, we explored the potential driver events for CRC pathogenesis through systematic analysis of genome‐wide RNA expression profiles and identified dysfunction of the cell cycle machinery as a vulnerability to oxaliplatin‐based chemotherapy in CRC. We demonstrate that PLK1 is significantly overexpressed in CRC tumors and that hyperactivity of PLK1 signaling is associated with poor prognosis. We show that pharmacological and genetic inhibition of PLK1 remarkably enhances the anti‐tumor effect of oxaliplatin in vitro and in vivo. Additionally, our results indicate that this combined effect of the PLK1 inhibitor and oxaliplatin was mediated by inhibition of the MYC‐CDC7 axis. Moreover, our data revealed an important role of the PLK1‐MYC‐CDC7 axis in chemoresistance and targeting this axis may provide a novel therapeutic strategy to overcome resistance to oxaliplatin‐based chemotherapy.

Although PLK1 has been proven oncogenic in various cancers,^[^ ^4^, ^14^ ^]^ its role in colon cancer is still controversial.^[^ ^15^ ^]^ High PLK1 expression has been associated with better OS, while IHC analysis demonstrated the opposite result, which showed that a high PLK1 score predicted worse OS.^[^ ^11^, ^12^, ^13^ ^]^ A recent study showed that PLK1 had tumor‐suppressive potential in colon cancer cells and that PLK1 overexpression increased the survival of colon cancer patients.^[^ ^15^ ^]^ However, our previous study indicated that PLK1 overexpression was correlated with oncogenic transformation and CSC self‐renewal.^[^ ^14^ ^]^ In this study, we further demonstrated that aberrant activation of the PLK1 signaling pathway was associated with poor prognosis and tumor recurrence in CRC. Although recent studies indicated that overexpression of PLK1 was found in CRC, few of them showed that dysregulation of PLK1 signaling is associated with chemoresistance. Our study indicated that hyperactivity of PLK1 was significantly increased in relapsed/metastatic CRC, providing new insights that PLK1‐coupled machinery was also associated with metastasis of CRC. In addition, we showed that the inhibition of PLK1 significantly sensitizes CRC cells to oxaliplatin‐based chemotherapy and combination treatment with a PLK1 inhibitor and oxaliplatin exhibits a synergistic effect in CRC in vitro and in vivo. Consistent with our findings, PLK1 inhibitors were shown to exhibit a synergistic effect with conventional chemotherapy drugs in various cancer types, suggesting the potential therapeutic value of PLK1 inhibitors to reverse chemoresistance in CRC treatment.^[^ ^34^, ^35^, ^36^ ^]^

Recently, an increasing number of clinical trials have focused on evaluating anti‐tumor effect of PLK1 inhibitors in patients with relapsed or refractory cancers.^[^ ^8^, ^18^ ^]^ However, the pharmacological mechanisms by which PLK1 inhibition revers chemoresistance remain unclear. The diverse effect of PLK1 inhibition in cancer therapy motivates us to identify the downstream effector as an attractive target.^[^ ^15^, ^16^ ^]^ Using genome‐wide expression profiling analysis, we showed that PLK1 inhibition suppressed a cluster of cell cycle‐related genes that were upregulated by oxaliplatin treatment. Among these genes, CDC7 was the top candidate gene whose overexpression was associated with a poor CRC prognosis. A previous study showed that CDC7 overexpression was an independent marker for good prognosis in CRC, which was inconsistent with studies on other cancer types where CDC7 overexpression was associated with worse prognosis.^[^ ^37^ ^]^ For example, high CDC7 expression correlated with poor prognosis in hepatocellular carcinoma,^[^ ^32^ ^]^ epithelial ovarian carcinoma,^[^ ^33^ ^]^ diffuse large B‐cell lymphoma^[^ ^38^ ^]^ and oral squamous cell carcinoma.^[^ ^39^ ^]^ In this study, we found that CDC7 overexpression was not only associated with poor prognosis, but also with tumor relapse/metastasis in CRC, and one possible explanation may be due to different therapeutic strategies in these cohorts.

**Figure 7:**
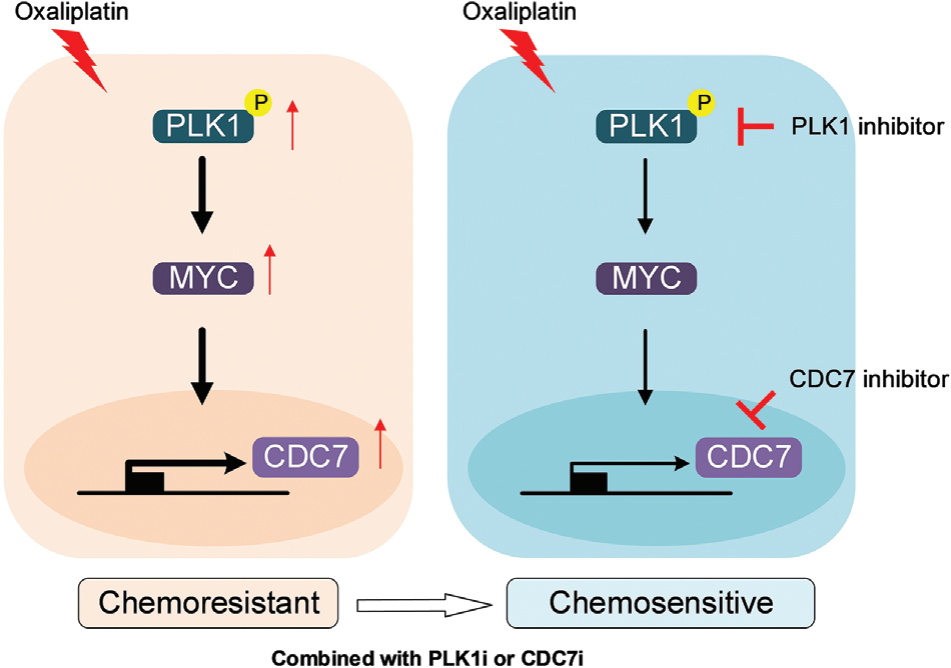
Schematic model illustrating the role of PLK1‐MYC‐CDC7 axis in mediating oxaliplatin resistance in CRC. Hyperactivity of PLK1‐MYC‐CDC7 axis was correlated with the resistance to oxaliplatin (left). Pharmacological inhibition of PLK1 dramatically suppressed CDC7 expression via MYC transactivation, resulting in sensitization to oxaliplatin treatment (right). Targeting PLK1‐MYC‐CDC7 axis could be an attractive therapeutic strategy to improve the clinical outcomes of oxaliplatin‐based chemotherapy in CRC.

CDC7 belongs to the serine‐threonine kinase family, is transcriptionally regulated by E2F1,^[^ ^40^, ^41^ ^]^ and is required for the initiation of DNA replication through S phase.^[^ ^33^ ^]^ The anti‐tumor effects of CDC7 inhibition have been reported in various cancers and orally bioavailable CDC7 inhibitors have been created for clinical trials.^[^ ^42^ ^]^ We further identified CDC7 as a key downstream effector of PLK1/MYC signaling. In line with our findings, recent studies have shown that CDC7 is a potential downstream target of MYC.^[^ ^43^, ^44^ ^]^ However, none of these studies demonstrated how MYC regulates CDC7 in detail, whereas the role of the MYC‐CDC7 axis remains unclear. Pérez‐Olivares M et al. detected a significant decrease in the level of CDC7 protein in MYC‐deficient B lymphocytes.^[^ ^43^ ^]^ However, they did not find any differences in CDC7 mRNA level through RNA‐seq. Hence, they speculated that the decrease in CDC7 protein might be due to the post‐transcriptional regulation of MYC.^[^ ^43^ ^]^ Another study showed that treatment with ETC‐159, a Wnt/beta‐catenin inhibitor, reduced both MYC and CDC7 mRNA levels.^[^ ^45^ ^]^ However, they noted that CDC7 may be an MYC‐independent gene. We now show that MYC can bind to the proximal promoter region of CDC7 and regulate its transcription. We further demonstrated that PLK1 regulates CDC7 activity in an MYC‐dependent manner. In addition, our study demonstrated that pharmacological targeting of the downstream effector of PLK1/MYC overcomes resistance to oxaliplatin, thus highlighting the pharmacological value of CDC7 inhibitors in PLK1/MYC driven tumors (**Figure** **7**). Indeed, combination treatment with a CDC7 inhibitor and oxaliplatin achieved a strong synergistic effect in vitro and in vivo, suggesting the potential clinical value of a CDC7 inhibitor in CRC. Our pre‐clinical study provides strong evidence of combination treatment with a CDC7 inhibitor and oxaliplatin for clinical use in CRC.

## 4 Conclusion

In summary, our study demonstrated the pharmacological value of PLK1 inhibitors as therapeutic candidates for CRC. CDC7 is a key factor underlying the mechanism of combination treatment with PLK1 inhibitors and oxaliplatin. Pharmacological targeting of the PLK1‐MYC‐CDC7 axis enhanced the efficacy of oxaliplatin, which provided potential clinical advantage in using PLK1 or CDC7 inhibitor in combination with chemotherapy regimens in treatment of CRC patients.

## 5 Experimental Section

### Clinical Samples and Tissue Microarrays

In this study, paraffin‐embedded pathological specimens were obtained from 343 patients with CRC who underwent initial resection, which were collected from January 2002 to June 2006 with a 10 year follow‐up. The clinicopathological variables collected from the CRC database of the follow‐up office included general information, surgical details, surgical techniques, tumor location, degree of differentiation, depth of tumor invasion, nodal status, metastatic status, and outcome follow‐up data. Tumor pT, pN, and pM status was assessed according to the criteria of the Seventh Edition of the American Joint Committee on Cancer (AJCC) stage.^[^ ^46^ ^]^ The collection of clinicopathological data from the enrolled patients from the CRC database was approved by the Institutional Review Board (IRB) of the Sixth Affiliated Hospital, Sun Yat‐sen University (approval number: 2020ZSLYEC‐198). The primary endpoint was OS, defined as the time interval from diagnosis to death.

The pathological specimens were constructed in TMAs. In the TMA construction, the central part of each primary tumor was identified by two senior pathologists, and then two cores (1 mm diameter each) were punched from these representative tumor areas and deposited into a recipient block at defined positions using a tissue array instrument (Beecher Instruments, Alphelys, France). The recipient blocks were subsequently cut into 5‐µm sections for IHC staining. TMA slides were deparaffinized and rehydrated through graded alcohols before being exposed to an antigen retrieval system (10 mm sodium citrate, 0.05% Tween‐20, pH 6.0 for 25 min). Endogenous peroxidase was blocked with 0.3% hydrogen peroxide for 10 min at room temperature. Then, the sections were incubated with the primary antibody overnight at 4 °C and stained with diaminobenzidine. The antibodies used in this study are provided in Table S3, Supporting Information.

### Evaluation of Immunohistochemical Analysis

Immunoreactivity for IHC staining was evaluated by a semiquantitative method, as described previously.^[^ ^47^ ^]^ Each TMA spot was assigned an intensity score from 1 to 4 (1, 2, 3, or 4) by two trained researchers. The percentage of positive tumor cells divided by the total number of tumor cells was assigned using 25% increments (25%, 50%, 75%, and 100%). IHC scores were determined by the intensity score and the proportion of area positively stained tumor cells. A final score was determined as the average of two cores from the same representative tumor area.

### Cell Lines and Drug Treatment

All cancer cell lines were purchased from the American Type Culture Collection (Manassas, VA) and were authenticated with short tandem repeat analysis. Cells were grown in DMEM or RPMI (Invitrogen) with 10% fetal bovine serum (HyClone, GE Healthcare Life Sciences) and 1% penicillin‐streptomycin (Gibco) and maintained at 37 °C in a 5% CO_2_ incubator. Mycoplasma contamination in cell culture was routinely tested every month. BI2536 and oxaliplatin were purchased from Selleck (Houston, Texas). GSK461364, volasertib, XL413, and PHA767491 were purchased from Topscience (Shanghai, China).

For the colony formation assay, single‐cell suspensions were plated (20 000 cells per well) in 6‐well plates, and the medium was refreshed every 3 days for 12 days of culture. Colonies were fixed in methanol, stained with crystal violet (0.5% crystal violet, 20% methanol), and photographed. For the cell viability assay, cells were seeded in 96‐well plates at the optimal seeding density in triplicate. After 24 h, cells were treated with different concentrations of drugs and cultured at 37 °C for 96 h, and the number of viable cells was measured by using the CellTiter‐Glo Luminescent Cell Viability Assay (#G7573, Promega, Madison, WI) according to the manufacturer's instructions. For the tumorsphere formation assay, single‐cell suspensions were plated (5000 cells per well) in 12‐well ultralow attachment plates with Mammocult medium (STEMCELL Technologies, Vancouver, BC) supplemented with fresh hydrocortisone (0.5 µg mL^−1^) and heparin (1:500). Cells were cultured for 10 days to form spheres and to be photographed.

### Flow Cytometric Analysis

Cells were treated with drugs for the indicated times and then harvested and fixed in 70% ethanol. Fixed cells were stained with Alexa Fluor647‐conjugated p‐H3 (S28) (Biolegend, 641006) and propidium iodide. The stained cells were analyzed for DNA content using an SP6800 flow cytometry (Sony Biotechnology, Tokyo, Japan).

### Plasmid Constructs and Inducible Cell lines

Full‐length PLK1 and MYC were amplified by RT‐PCR using 1000 ng of total RNA from HEK293 cells and the following primers: PLK1‐F: 5′‐ATGAGTGCTGCAGTGACTGC‐3′ and PLK1‐R 5′‐TTAGGAGGCCTTGAGACGGT‐3′; MYC‐F: 5′‐ATGGATTTTTTTCGGGTAGT‐3′ and MYC‐R: 5′‐TTACGCACAAGAGTTCCGT‐3′. To establish of overexpressed stable cells, the PCR products were cloned into pCDH‐EF1‐MCS‐T2A‐Puro (System 331 Bioscience, Palo Alto, CA, USA) vectors for lentiviral infection. In brief, the lentiviral expression plasmid, packaging plasmid psPAX.2 (Addgene, Cat# 12260), and VSV‐G envelope expressing plasmid pMD2.G (Addgene, Cat# 12259) were co‐transfected into HEK293T cells. After 48 h, the lentiviruses were harvested to infect cancer cells and then selected with puromycin (2 µg mL^−1^) (Thermo Fisher Scientific) for 4 days.

### Western Blotting

Protein extracts were prepared with RIPA cell lysis buffer (150 mm NaCl, 50 mm Tris‐HCl, 0.5% deoxychlorate sodium, 200 mm NaF, 200 mm PMSF, 1.0% NP40, and 1 mm EDTA) with a protease inhibitor cocktail (Roche, Basel, Switzerland). Lysates and prestained protein marker (M221, GenStar, Beijing, China) were subjected to SDS‐PAGE and transferred to PVDF membranes for immunoblotting analysis. The antibodies used in this study are provided in Table S3, Supporting Information.

### RNA Interference

The siRNA and the nontargeting control were purchased from RiboBio (Guangzhou RiboBio, CO). Cells were transfected with 100 nm final concentration of siRNA duplexes using Lipofectamine RNAiMAX (Life Technologies, Gaithersburg, MD) following the manufacturer's instructions. The siRNA sequences are provided in Table S4, Supporting Information.

### Real‐Time Quantitative PCR

Total RNA was extracted from cells using an RNeasy Mini Kit (QIAGEN, Duesseldorf, Germany) according to the manufacturer's protocol. One microgram of RNA was used for RT‐qPCR using a reverse transcription kit (#AT341‐02, Transgen Biotech, Beijing, China) and quantitative kit (#N30920, Transgen Biotech, Beijing, China) on a Biorad CFX Real‐time PCR machine. All qPCR experiments were performed in triplicate, and mean values were used to determine mRNA levels. Relative quantification was performed using the comparative CT method with 18S as the reference gene and with the formula 2‐ΔΔCT. Error bars showed standard deviation. The primer sequences are listed in Table S5, Supporting Information.

### ChIP‐Quantitative PCR

Transcription factor ChIP was performed with 10^7^ cells per reaction. An EZ‐Magna ChIP A/G Chromatin Immunoprecipitation Kit (Millipore, 17–10086) was used, followed by the manufacturer's protocol. Cells were crosslinked in 1% formaldehyde, quenched in 0.125 m glycine and lysed in cell lysis buffer (50 mm Tris pH 8.1, 10 mm EDTA, 1% SDS). Lysates were sonicated with Bioruptor Pico (Diagenode, Belgium) and diluted in dilution buffer containing protease inhibitors with an antibody against MYC (ab56, Abcam) or species‐matched IgG (12‐371, Millipore) and protein A/G magnetic beads. After overnight incubation, the samples were washed with three kinds of washing buffer one time with TE buffer. DNA was eluted in elution buffer at 62 °C for 2 h with shaking and purified with spin columns.RT‐qPCR was performed using the primers listed in Table S5, Supporting Information.

### Luciferase Assay

The promoter of CDC7 was cloned into the pGL3‐basic vector (Promega). All constructs were verified by Sanger DNA sequencing. Reporter plasmids were transfected into DLD1 or WiDr cells infected with siNC or siMYC for 48 h using Lipofectamine 3000 (Life Technologies, Gaithersburg, MD). Luciferase activities were analyzed using the Dual‐Luciferase Reporter Assay System (catalogue E1910, Promega). The sequence of the promoter of CDC7 was as follow: sense primer (5′‐3′): ATCCGTTATTGTCATCGCTTC; antisense primer (5′‐3′): GCAAGTTTAAAATTCTGCTCGTT.

### RNA‐Sequencing

Total RNA was extracted using the RNAeasy Mini Kit according to the manufacturer's protocol (QIAGEN, Duesseldorf, Germany). The mRNA libraries were prepared using a TruSeq Stranded Total RNA Sample Preparation kit with Ribo Zero Gold (Illumina), followed by sequencing on a NovaSeq sequencer (Illumina).

### Bioinformatics Analyses

For RNA‐seq analyses, raw counts were filtered and trimmed by fastp version 0.12.5 for clean data with default settings.^[^ ^48^ ^]^ The clean data were mapped to the human reference genome (GRCh38, hg38) using STAR aligner (version 2.7.0f), and the abundance of transcripts was quantified by RSEM.^[^ ^49^, ^50^ ^]^ Differential expression analyses were performed using DEseq2 (v3.14.0) (|log_2_FC| ≥ 1 and adjusted *p*‐value ≤ 0.05) in the R statistical environment (v3.5).^[^ ^51^ ^]^ GSEA was performed using GSEA software (v4.0) using the PID subsets and HALLMARK gene sets, and visualizations were conducted in the R statistical environment (v3.5).^[^ ^52^ ^]^

For ChIP‐seq analyses, data used in this study were available for download from the Gene Expression Omnibus (GEO) (http://www.ncbi.nlm.nih.gov/geo). The clean data were obtained from fastp and mapped to the human reference genome (GRCh38, hg38) using Bowtie2 (version 2.3.2) with default settings.^[^ ^53^ ^]^ The alignments were sorted and indexed using samtools version 1.^[^ ^54^ ^]^ Bigwig files for visualization were produced by Deeptools and Integrative Genomics Viewer.^[^ ^55^, ^56^ ^]^

### Accession Number

Illumina gene expression data of human CRC and matched normal controls can be found in the GEO archive under accession numbers GSE10972 and GSE74604.

CRC data to evaluate PLK1 expression in paired tumor and normal tissue were generated by GSE39582 (CIT cohort), TCGA (COADREAD), GSE20842 (Gaedcke Rectum), and GSE6988 (Dong Colorectum). The CHIP‐seq data with antibodies specific for MYC were generated from GSE36354. RNA‐seq data have been deposited in the GEO database under 160089.

### Organoid Culture

Colorectal tumor tissues were obtained from The Sixth Affiliated Hospital of Sun Yat‐sen University with informed consent and the study was approved by the ethical committee (SYSU‐IACUC‐2020‐000570). Fresh tumor tissues were dissociated into single‐cell suspensions with the Tumor Dissociation Kit (Miltenyi Biotec, 130‐095‐929). CRC organoids were cultured in CRC organoid medium.^[^ ^57^ ^]^ The composition of CRC organoid medium was: 14 mL advanced DMEM/F12 medium (Life Technologies) with 1% GlutaMax I 100x (Life Technologies), 1% Hepes (Life Technologies), 1% Penicillin Streptomycin 100x (Thermofisher), 0.1% Primocin (Invivogen), 2% B27 supplement 50x (Life Technologies), 3.5 µg R‐Spondin 3 (R&D), 2.8 µg EGF (Peprotech), 2.95 µg A83‐01 (Tocris), 1.4 µg Wnt3A (R&D), 46.4 µg SB202190 (Sigma), 2.24 mg *N*‐acetylcysteine (Sigma), 0.168 mg nicotinamide (Sigma), and 1% N2 supplement 100x (Life Technologies).

### Mice Experiments

Female athymice BALB/c nude mice (5–8 weeks old) were purchased from Charies River (Beijing, China) and housed in the Biological Resource Centre. Mice were implanted subcutaneously in the flank with 5 × 10^6^ DLD1 cells in 0.1 mL PBS. When tumors reached 70 mm^3^, randomization was performed by equally dividing tumor‐bearing mice of similar tumor burden into control and drug treatment groups: treatment with DMSO (*n* = 11, i.p.), volasertib (*n* = 10, 10 mg kg^−1^, every 3 days, i.p.), oxaliplatin (*n* = 10, 3 mg kg^−1^, every 3 days, i.p.), and a combination in which each compound was administered at the same dose and schedule as the single agent (*n* = 10, i.p.). Tumor progress was monitored with whole body weight and tumor size every other day. Tumors were measured by Vernier calipers and calculated with the following formula: *V* = *W* ^2^ × *L*/2. No experimental samples were excluded in this study, with one mouse died from an unexpected illness in the combination treatment group. Following drug treatment, the tumors were monitored for ≈3 weeks. Tumors were excised upon reaching 1000 mm^3^ or at the end of the study. All animal studies were conducted in compliance with animal protocols approved by the Animal Care and Ethics Committee of Sun Yat‐sen University and followed the National Health Guidelines on the Care and Use of Animals (SYSU‐IACUC‐2020‐000191).

For the pharmaceutical experiments of XL413, mice of similar tumor burden were divided into four groups: treatment with 0.9% NaCl (*n* = 10, oral gavage), XL413 (*n* = 10, 20 mg kg^−1^, once a day, oral gavage), oxaliplatin (*n* = 9, 3 mg kg^−1^, every 3 day, i.p.), and a combination in which each compound was administered at the same dose and schedule as the single agent (*n* = 10).

For the establishment of a PDX model, female NOD/MrkBomTac‐Prkdc^scid^ (NOD‐SCID) mice (5–8 weeks old) were purchased from Charies River (Beijing, China) and housed in the Biological Resource Centre. Colorectal tumor tissues were obtained from The Sixth Affiliated Hospital of Sun Yat‐sen University with informed consent, and the study was approved by the ethical committee (SYSU‐IACUC‐2020‐000570). Mice were implanted subcutaneously with pieces of fresh tumor from whole‐tumor just dividing from CRC patients in surgery. The tumors were washed in PBS and transported to DMEM/F12 basic medium. Whole tumors were minced with a sterile scalpel to patches that could pass through a needle bore, and then injected into the mice subcutaneously. Randomization was performed by equally dividing tumor‐bearing mice of similar tumor burden into control and experimental groups for drug treatment. Tumors were excised upon reaching 1500 mm^3^ or at the end of the study.

### Statistical Analysis

Data were presented as the mean ± SD, unless otherwise stated. Statistical significance between two groups was evaluated by two‐tailed Student's *t*‐test, while statistical significance among multiple groups was analyzed by two‐way ANOVA with Dunnett's multiple comparisons test using GraphPad Prism software. Statistical significance was considered at *p* < 0.05.

## Conflict of Interest

The authors declare no conflict of interest.

## Author Contributions

Z.Y., P.D., and Y.C. contributed equally to this work. Z.Y., P.D., Y.C., P.L., J.T., and X.W. conceived, designed, and supervised the study; Z.Y. performed the experiments; P.D. and J.C. analyzed and interpreted the data (e.g., statistical analysis, biostatistics, and computational analysis); Y.C., Y.Z., Z.C., D.L., and X.W. provided patient samples; X.F., Y.H., and P.H. provided pathology expertise; Z.Y. and Y.C. performed the tissue array. S.L. performed the ChIP experiments. Z.Y., P.D., P.W., R.X., J.C., Y.W., and J.C. provided material and technical support and animal work; Z.Y. and J.T. wrote, reviewed, and revised the manuscript. Q.Y., P.L., and X.W. provided critical readings of the manuscript. All the authors have given their consent to publish this study.

## Supporting information

Supporting Information

## Data Availability

The data that support the findings of this study are available: illumina gene expression data of human CRC and matched normal controls can be found in GEO archive under accession number GSE10972 and GSE74604. Colorectal cancer data to evaluate PLK1 expression in paired tumor and normal tissue were generated by GSE39582 (CIT cohort), TCGA (COADREAD), GSE20842 (Gaedcke Rectum), and GSE6988 (Dong Colorectum). The data of CHIP‐seq with antibodies specific for MYC was generated from GSE36354. RNA‐seq data have been deposited in GEO database under 160089.
