# Supplementary material for: Inhibition of the PLK1‐Coupled Cell Cycle Machinery Overcomes Resistance to Oxaliplatin in Colorectal Cancer
Source: Adv Sci (Weinh). 2021 Oct 28;8(23):2100759. doi: 10.1002/advs.202100759 (PMC8655181; doi:10.1002/advs.202100759)
Supplement: Supplementary file 1 — Supporting Information [file ADVS-8-2100759-s001.pdf]

© 2021 Wiley-VCH GmbH

ADVANCED SCIENCE
Open Access

Supporting Information

for *Adv. Sci.,* DOI: 10.1002/advs.202100759

Targeting dysregulated PLK1-coupled cell cycle machinery overcomes the resistance to oxaliplatin in colorectal cancer

*Zhaoliang Yu[1,] #, Peng Deng[2,] #, Yufeng Chen[1,] #, Shini Liu[2], Jinghong Chen[2], Zihuan Yang[3], Jianfeng Chen[2], Xinjuan Fan[4], Peili Wang[2], Zerong Cai[1], Yali Wang[2], Peishan Hu[3], Dezheng Lin[5], Rong Xiao[6], Yifeng Zou[1], Yan Huang[4], Qiang Yu[7, 8], Ping Lan[1, 3] *, Jing Tan[2, 9] *, Xiaojian Wu[1, 3] **

Targeting dysregulated PLK1-coupled cell cycle machinery overcomes the resistance to oxaliplatin in colorectal cancer

Short Title: Targeting PLK1-MYC-CDC7 axis in CRC.

Zhaoliang Yu[1,] #, Peng Deng[2,] #, Yufeng Chen[1,] #, Shini Liu[2], Jinghong Chen[2], Zihuan Yang[3], Jianfeng Chen[2], Xinjuan Fan[4], Peili Wang[2], Zerong Cai[1], Yali Wang[2], Peishan Hu[3], Dezheng Lin[5], Rong Xiao[6], Yifeng Zou[1], Yan Huang[4], Qiang Yu[7, 8], Ping Lan[1, 3] *, Jing Tan[2, 9] *, Xiaojian Wu[1, 3] *

This supporting information includes:

Five supplementary tables.

Supplementary Tables

Supplementary Table 1. Patient characteristics of the tissue microarray.

Characteristic		All cases
Number of patients		343
Age at the time of surgery, yrs		59.44± 11.56
Male patients, n (%)		186 (54.2%)
Pathological T stage, n (%)		
	pT0 or pT1 or pT2	52 (15.2%)
	pT3 or pT4	291 (84.8%)
Pathological N stage, n (%)		
	pN0	187 (54.5%)
	pN1 or pN2	156 (45.5%)
Pathological M stage, n (%)		
	pM0	328 (95.6%)
	pM1	15 (4.4%)
Pathological TNM stage, n (%)		
	pTNM0 or pTNM1 or pTNM2	186 (54.2%)
	pTNM3 or pTNM4	157 (45.8%)
Histopathology, n (%)		
	Adenocarcinoma	339 (98.8%)
	Others	4 (1.2%)
Differentiation, n (%)		
	Well	103 (30.0%)
	Moderate or poor	240 (70.0%)
Adjuvant chemotherapy, n (%)		210 (61.2%)

Median follow-up time, yrs	6.17

Supplementary Table 2. Univariate and multivariate analyses showing that PLK1 expression could serve as an independent prognostic factor for the recurrence of CRC.

Variables	Univariate Analysis		Multivariate Analysis	
	RR (95% CI)	P-value	RR (95% CI)	P-value
Age (≤60 years vs. >60 years)	1.14 (0.70-1.87)	0.592		
Gender (male vs. female)	1.10 (0.67-1.80)	0.715		
Pathological T stage (pT0-2 vs. pT3-4)	3.93 (1.51-10.23)	0.005		
Pathological N stage (pN0 vs. pN1-2)	2.99 (1.79-4.98)	<0.001		
Pathological TNM stage (pTNM0-2 vs. pTNM3)	3.14 (1.88-5.24)	<0.001	2.73 (1.54-4.83)	0.001
Differentiation (well vs. moderate/poor)	1.17 (0.68-2.01)	0.578		
PLK1 expression (low vs. high)	3.13 (1.69-5.78)	<0.001	2.77 (1.46-5.28)	0.002
p-PLK1 expression (low vs. high)	2.47 (1.37-4.42)	0.002		

Supplementary Table 3. List of antibodies used in the study.

Antibodies used in western blot				
Cat. No	Antibody	Species	dilution	Company
ab17056	PLK1	Mouse	1:2000	Abcam
ab155095	p-PLK1 (T210)	Rabbit	1:1000	Abcam
ab32072	MYC	Rabbit	1:1000	Abcam
sc-56275	CDC7	Mouse	1:200	Santa Cruz
ab108935	MCM2	Rabbit	1:2000	Abcam
ab133243	p-MCM2 (S40)	Rabbit	1:500	Abcam
ab124707	Dbf4	Rabbit	1:10000	Abcam
Ab32107	p-H3 (S10+T11)	Rabbit	1:2000	Abcam
2118S	GAPDH	Rabbit	1:5000	CST

Antibodies used in IHC				
Cat. No	Antibody	Species	dilution	Company
ab17056	PLK1	Mouse	1:400	Abcam
ab155095	p-PLK1 (T210)	Rabbit	1:400	Abcam
ab32072	MYC	Rabbit	1:100	Abcam
sc-56275	CDC7	Mouse	1:25	Santa Cruz
#9661	Cleaved Caspase 3	Rabbit	1:100	CST
ZA-0502	Ki-67	Rabbit	1:1000	ZSGB-BIO

Supplementary Table 4. The sequences of siRNAs.

**siRNA sequence**			
Name	Target gene	Gene ID	Target sequence
si*PLK1* #1	*PLK1*	5347	CAACCAAAGTCGAATATGA
si*PLK1* #3	*PLK1*	5347	CCTTAAATATTTCCGCAAT
si*MYC* #1	*MYC*	4609	GAGGAGACATGGTGAACCA
si*MYC* #3	*MYC*	4609	CGACGAGACCTTCATCAAA
si*CDC7*	*CDC7*	8317	cgTGATGTTAAGCCCAGCAAT

Supplementary Table 5. List of quantitative real-time PCR primers and ChIP PCR primers.

**qPCR sequence**		
Target gene	Sense Primer (5'-3')	Antisense Primer (5'-3')
CDC7	GAGGCGTCTTTGGGGATTCAG	GGTCCTACTTGTAACTGTGCTG
NCAPG2	TACAAGCCGTGTCTAAGGAGC	TTGAGCCATGTTCGGTTTCCA
SMC1A	AACCTGCGGGTAAAGACCCT	GGCAAAGGTACGGTCCTCAG
MELK	TCTCCCAGTAGCATTCTGCTT	TGATCCAGGGATGGTTCAATAGA
HELLS	GAGGCTCCAGCAATGGTTGAA	CGCTCTCTCTCTAGTCCAGCA
KIF23	AGTCAGCGAGAGCTAAGACAC	GGTTGAGTCTGTAGCCCTCAG
TOP2A	ACCATTGCAGCCTGTAAATGA	GGGCGGAGCAAAATATGTTCC
FANCD2	AAAACGGGAGAGAGTCAGAATCA	ACGCTCACAAGACAAAAGGCA
POLA1	ACGCCAGGATGATGACTGGA	GTCACTGCGAGCTTCTTTACAT

**CHIP-qPCR sequence**			
Name	Target gene	Sense Primer (5'-3')	Antisense Primer (5'-3')
CDC7-P1	*CDC7*	CTGGATCGTGACTTCGGTTT	GAGGAGAAGGGTGAATGAATGG
CDC7-P2	*CDC7*	CCTTAGACAAGGCCACAGTATG	CTCGCTAAACCACGCACTAA
CDC7-P3	*CDC7*	GCGGGATTGTGAGGGATTAG	ACCGAACCAGATGCTTAGTG
CDC7-P4	*CDC7*	GATGTGTCAGCACCTGAGATT	GAATGAAGCCCGTGGATGAT
Control		CGGTATGGAGCCCTGAAGACT	AGGTGAGGGAGGTGGCTTAGA
